# Current status of cardiac rehabilitation among representative hospitals treating acute myocardial infarction in South Korea

**DOI:** 10.1371/journal.pone.0261072

**Published:** 2021-12-08

**Authors:** Myung Soo Park, Sunki Lee, Taehoon Ahn, Doyoung Kim, Mi-Hyang Jung, Jae Hyuk Choi, Seongwoo Han, Kyu Hyung Ryu, Eung Ju Kim

**Affiliations:** 1 Department of Cardiology, Hallym University Dongtan Sacred Hospital, Hwaseong, South Korea; 2 Department of Cardiology, Korea University Anam Hospital, Seoul, South Korea; 3 Department of Cardiology, Korea University Guro Hospital, Seoul, South Korea; Case Western Reserve University School of Medicine, UNITED STATES

## Abstract

Cardiac rehabilitation services are mostly underutilized despite the documentation of substantial morbidity and mortality benefits of cardiac rehabilitation post-acute myocardial infarction. To assess the implementation rate and barriers to cardiac rehabilitation in hospitals dealing with acute myocardial infarction in South Korea, between May and July 2016, questionnaires were emailed to cardiology directors of 93 hospitals in South Korea; all hospitals were certified institutes for coronary interventions. The questionnaires included 16 questions on the hospital type, cardiology practice, and implementation of cardiac rehabilitation. The obtained data were categorized into two groups based on the type of the hospital (secondary or tertiary) and statistically analysed. Of the 72 hospitals that responded (response rate of 77%), 39 (54%) were tertiary medical centers and 33 (46%) were secondary medical centers. All hospitals treated acute myocardial infarction patients and performed emergency percutaneous coronary intervention; 79% (57/72) of the hospitals performed coronary artery bypass grafting. However, the rate of implementation of cardiac rehabilitation was low overall (28%, 20/72 hospitals) and even lower in secondary medical centers (12%, 4/33 hospitals) than in tertiary centers (41%, 16/39 hospitals, *p* = 0.002). The major barriers to cardiac rehabilitation included the lack of staff (59%) and lack of space (33%). In contrast to the wide availability of acute-phase invasive treatment for AMI, the overall implementation of cardiac rehabilitation is extremely poor in South Korea. Considering the established benefits of cardiac rehabilitation in patients with acute myocardial infarction, more administrative support, such as increasing the fee for cardiac rehabilitation services by an appropriate level of health insurance coverage should be warranted.

## Introduction

Coronary heart disease (CHD) is a major cause of death and medical expenses in many developed countries [1, 2]. The number of patients with CHD is not only increasing, but CHD patients are also living longer with symptoms because of the major diagnostic and therapeutic developments for CHD in the last few decades [3]. Due to its increasing incidence, CHD has become a rising health problem and is now the second largest cause of death from a single organ disease in South Korea [4]. The rates of percutaneous coronary intervention (PCI) and coronary artery bypass graft (CABG) surgery have also increased markedly in accordance with this trend. Due to the widespread use of effective reperfusion therapy and optimal medical treatment, the mortality rate of CHD in South Korea has recently been on the decline, and the survivors need stronger strategies of recovery and secondary prevention [5].

Cardiac rehabilitation (CR) is defined by the World Health Organization as the “sum of activities required to influence favorably the underlying cause of the disease, as well as to provide the best possible physical, mental and social conditions, so that the patients may, by their own efforts, preserve or resume when lost, as normal a place as possible in the community” [6]. Many previous studies have shown that CR has numerous positive effects on patients with CHD, including a decrease of mortality. Hence, both the American Heart Association/American College of Cardiology (AHA/ACC) and European Society of Cardiology (ESC) guidelines include CR as a Class I recommendation [7, 8].

Despite the proven benefits of CR and guidelines promoting referral to CR, CR is not available in approximately 60% of countries worldwide; even in countries where CR is available, there are not enough services to meet the growing needs [9]. And only a few studies have characterized CR availability and implementation rates at national or regional levels [10, 11]. To date, there exists a scarcity of reports on the CR implementation rate in South Korea. In this study, we assessed the implementation rate and barriers of CR among the hospitals that were certified for coronary interventions and handling of AMI in South Korea.

## Materials and methods

From May to July 2016, we conducted a questionnaire-based survey of all 93 hospitals that were certified as institutes for coronary interventions by the Korean Society of Interventional Cardiology in South Korea. We sent an e-mail including 16 simple questions about CR to the cardiology department directors of each hospital. Since CR had been not widely activated in Korea, questions were focused on inpatient education and outpatient exercise rehabilitation program. With the National Health Insurance Service (NHIS) coverage, the CR program in Korea took shape and consisted of education and exercise for inpatient and exercise programs for outpatient up to 36 sessions. These program structures do not fit exactly into the phase divisions of CR, but roughly correspond to phases 1–3. To improve the response rate, we sent two reminders in the event of a non-response. The final response rate for the questionnaire was 77% (72/93 hospitals). This study received the local institutional review board of Korea University Guro Hospital. (NO. 2021GR0366)

The questionnaire consisted of 16 questions grouped under the following categories:

Hospital data: 1) Is it a secondary (general) or tertiary hospital? 2) Location of the hospital, 3) Number of hospital beds, 4) Number of cardiologists, 5) Presence of an exclusive coronary intensive care unit;

Cardiologic practice data: 6) Are AMI patients treated? 7) Is coronary angiography (CAG) performed? 8) Is percutaneous coronary intervention (PCI) performed? 9) Is emergent PCI performed for AMI patients? 10) Is coronary artery bypass graft (CABG) surgery performed?

Implementation of CR: 11) Is there a CR program in place? 12) What has been the biggest hurdle in your hospital for starting CR (barriers to CR)? If the hospital has been undertaking CR: 13) Is an educational program for AMI available? 14) Is an outpatient CR program available? 15) Is a cardiopulmonary exercise test available? 16) Is a cardiologist in-charge of the CR program?

Questions 2,3,4 and 12 were categorical questions and the rest were dichotomous. The data obtained from each hospital were categorized into two groups according to the hospital type (secondary or tertiary hospitals). Statistical analysis was performed to compare variables between the two groups. Numerical data are presented as means ± standard deviation. The chi-square test and Fisher’s exact test were used for categorical data to compare the rate of implementation of CR per the hospital grade. All tests were 2-tailed, and *p* < 0.05 was considered statistically significant.

## Results

**Fig 1:**
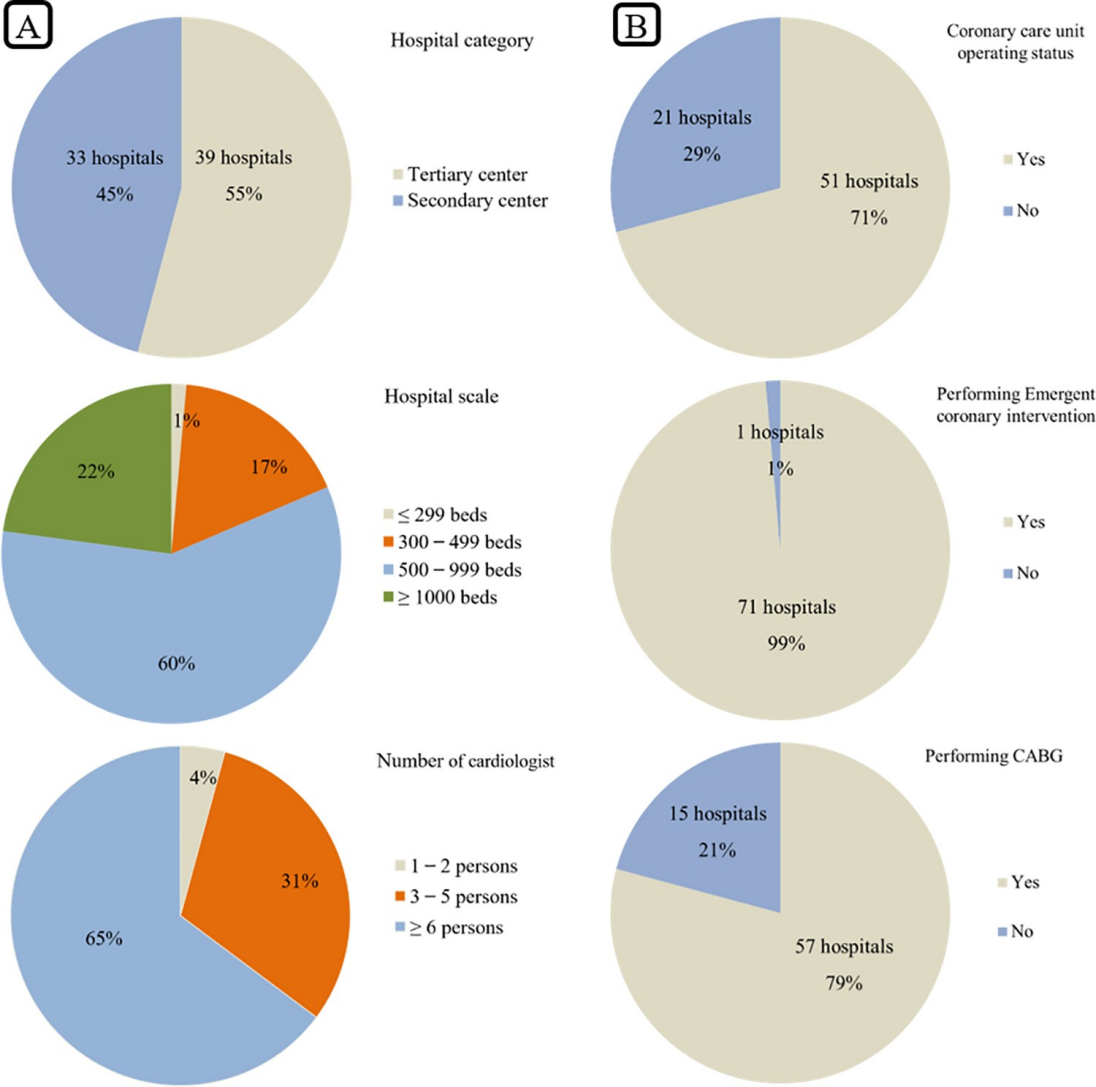
Pie charts showing hospital information and treatment of acute myocardial infarction. CABG = coronary artery bypass graft.

**Fig 2:**
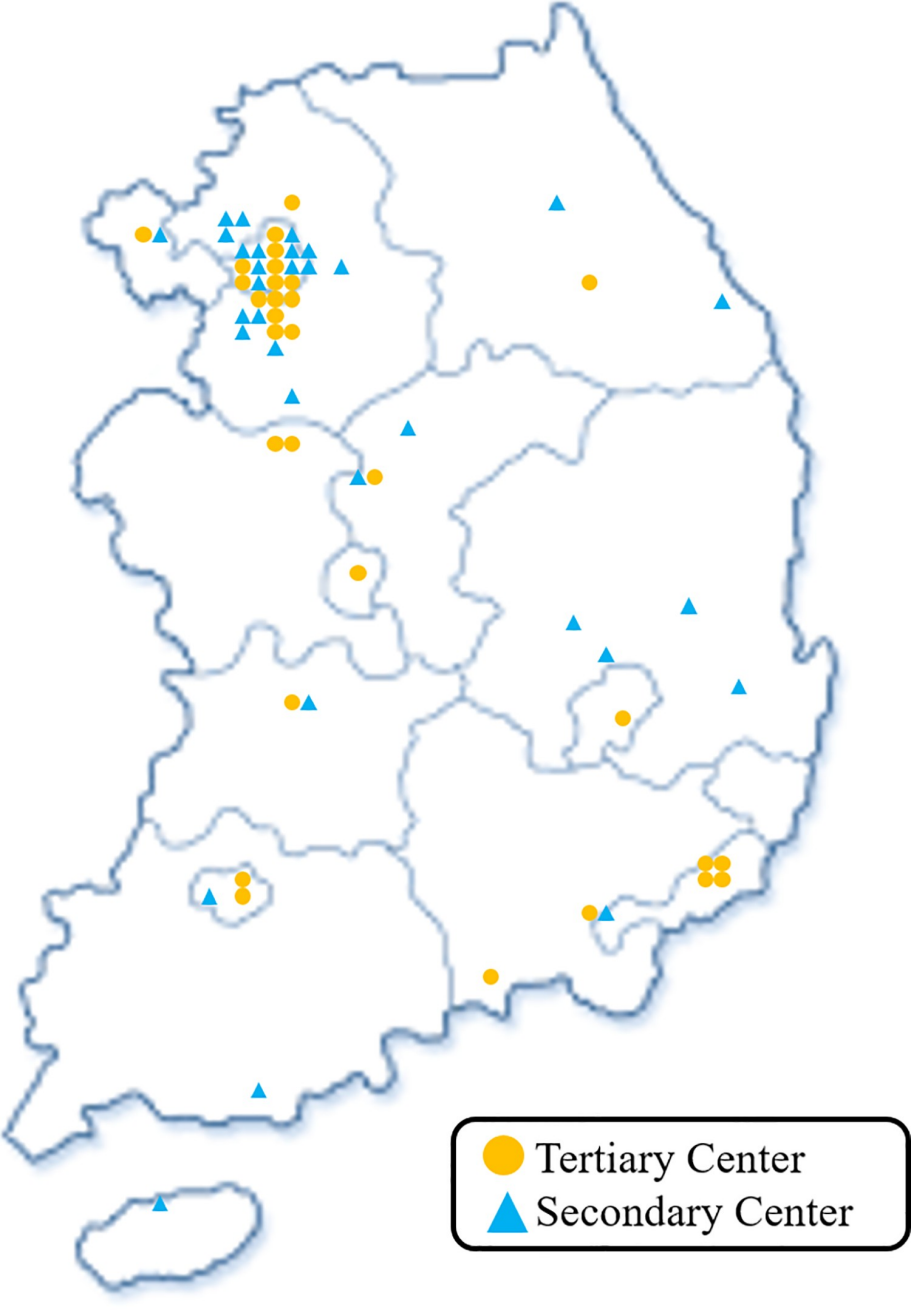
Geographical distribution of the responding hospitals.

The response rate was 77%, with 72 of 93 hospitals responding to the questionnaire-based survey. Data on the hospitals’ status are summarized in Fig 1A and Table 1. Of the 72 hospitals, 33 were secondary medical centers and 39 were tertiary medical centers. The hospitals that responded were located in various regions across the country. The administrative district of South Korea consists of one capital city (Seoul), six metropolitan cities (Busan, Daejeon, Daegu, Gwangju, Incheon, and Ulsan), eight provinces (Gyeonggi, Gangwon, North and South Chungcheoung, North and South Gyeongsang, and North and South Jeolla), and Jeju special self-governing province. The geographical distribution of the hospitals in our study was as follows (Fig 2): 23 hospitals in Seoul, 9 in metropolitan cities, 15 in Gyeonggi-do, 10 in North and South Gyeongsang-do, 6 in North and South Chungcheoung-do, 5 in North and South Jeolla-do, 3 in Gangwon-do, and 1 in Jeju Island. The scale of the hospitals was categorized according to the number of beds, representing their inpatient capacity. Hospitals with 500 to 999 beds were the most common among responding hospitals, accounting for 60% of the total. The difference in scale between tertiary and secondary hospitals was statistically significant, considering that large hospitals with 1000 or more beds were only in the tertiary hospital group (*p*<0.001). The number of cardiologists was six or more in 65% of hospitals. In the tertiary hospital group, 85% of hospitals had six or more cardiologists, while only 39% of secondary hospitals had six or more cardiologists; the majority (52%) of the secondary hospitals had between three to five cardiologists. There was also a statistically significant difference in the number of medical staff between the two groups (*p* < 0.001).

**Table 1:** Comparison of hospital data, acute myocardial infarction care, and implementation of cardiac rehabilitation between tertiary and secondary hospitals.

	Tertiary (n = 39)	Secondary (n = 33)	Total (n = 72)	P-value
Hospital data				
Number of hospital beds				<0.001
≥ 1000	16 (41%)	0 (0%)	16 (22%)	
500–999	22 (56%)	21 (64%)	43 (60%)	
300–499	1 (3%)	11 (33%)	12 (17%)	
≤ 299	0 (0%)	1 (3%)	1 (1%)	
Number of cardiologists				<0.001
≥ 6	33 (85%)	13 (39%)	46 (65%)	
3–5	6 (15%)	17 (52%)	23 (31%)	
1–2	0 (0%)	3 (9%)	3 (4%)	
Status of AMI care				
Performing CAG & PCI	39 (100%)	32 (99%)	71 (99%)	0.458
Emergent PCI	39 (100%)	32 (99%)	71 (99%)	0.458
CABG	38 (97%)	19 (58%)	57 (79%)	<0.001
Operating CCU	35 (90%)	16 (48%)	51 (71%)	<0.001
Implementation of CR	16 (41%)	4 (12%)	20 (28%)	<0.001

AMI = acute myocardial infarction; CABG = coronary artery bypass graft; CAG = coronary angiography; CCU = coronary care unit; CR = cardiac rehabilitation; PCI = percutaneous coronary intervention.

The AMI care data are summarized in Fig 1B and Table 1. Approximately 90% of tertiary hospitals and 48% of secondary hospitals had an exclusive coronary care unit; this difference was statistically significant (*p* < 0.001). All hospitals surveyed, except for one center, were able to perform elective CAG, PCI, and emergent PCI for AMI treatment. The one hospital that responded that coronary intervention was not possible had only temporarily disbanded these facilities. Thus, in essence, every center that replied to the survey had sufficient medical ability and systems in place for acute phase reperfusion treatment of AMI patients. CABG was available in 97% and 58% of tertiary and secondary hospitals, respectively, and this difference was statistically significant (*p* < 0.001).

**Fig 3:**
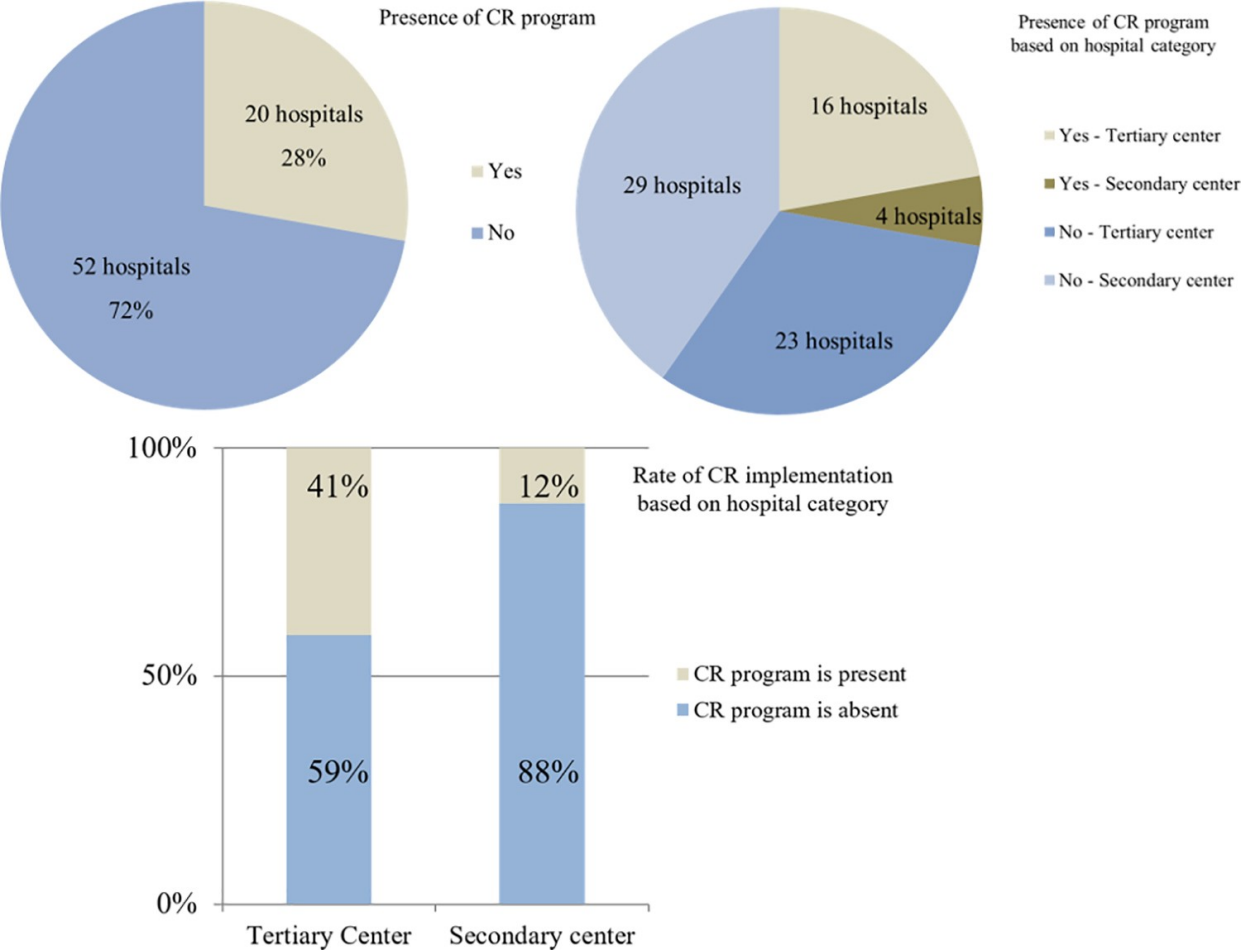
Status of implementation of cardiac rehabilitation. CR = cardiac rehabilitation.

**Fig 4:**
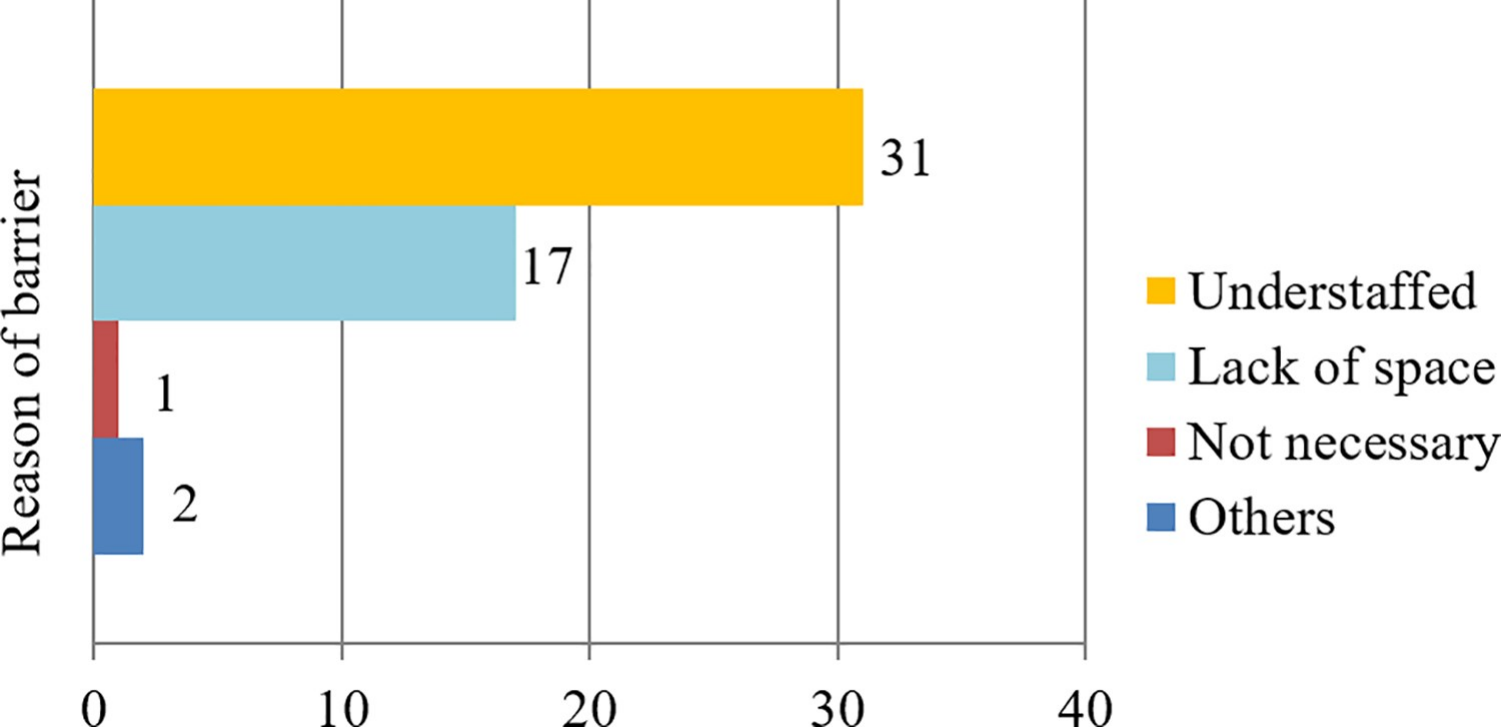
Major barriers to cardiac rehabilitation among the hospitals not running a rehabilitation program (n = 52).

**Fig 5:**
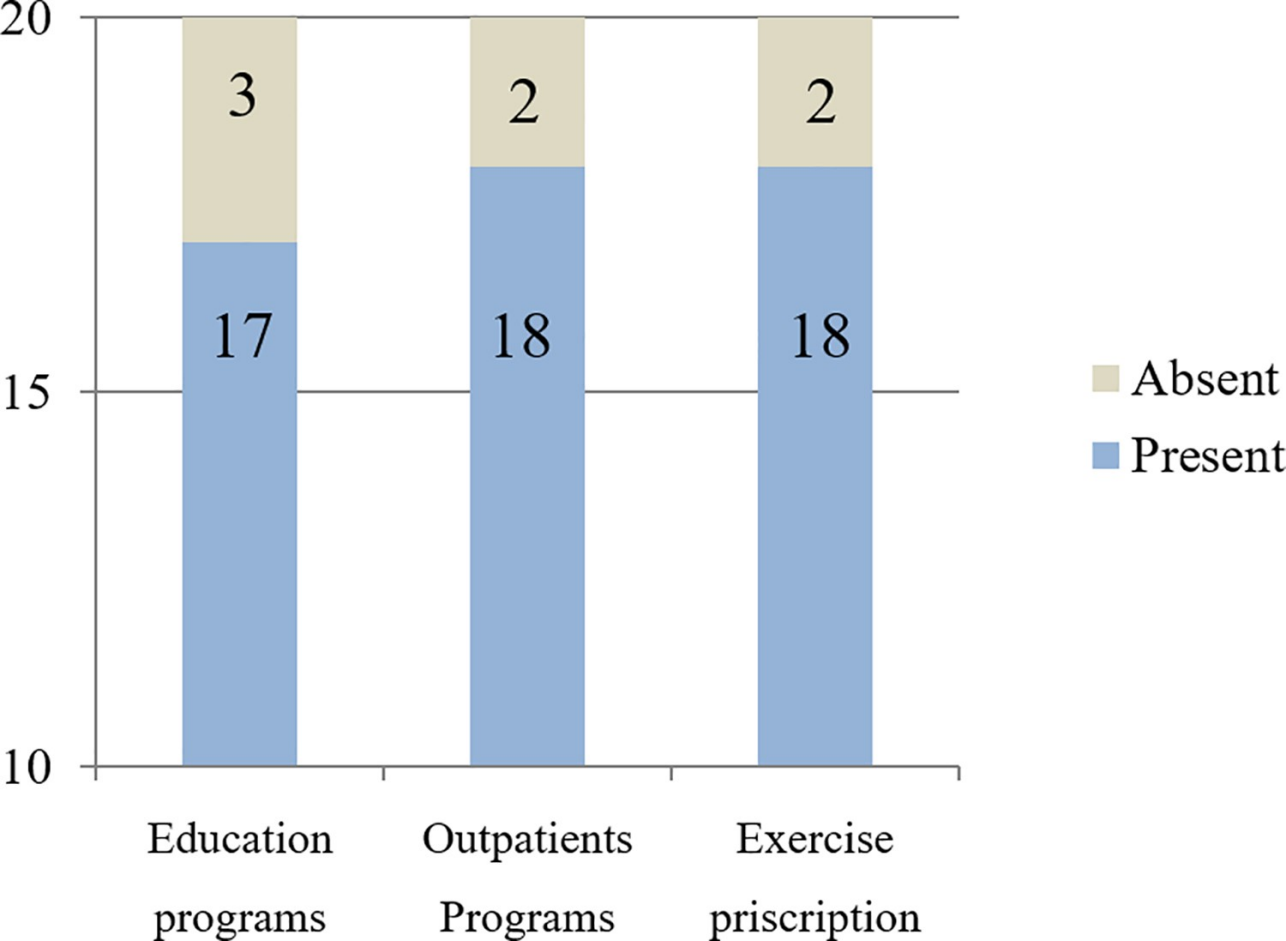
Details of programs in hospitals running cardiac rehabilitation (n = 20).

Data on the implementation of CR in hospitals in South Korea are summarized in Fig 3. Of the 72 hospitals that responded to the questionnaire, 20 (28%) had CR programs and 52 did not. Among the 20 hospitals with CR programs, 16 (80%) were tertiary centers; among the 52 hospitals without a CR program, 29 hospitals (56%) were secondary centers. The CR implementation rate varied based on the hospital category: 41% in tertiary hospitals (16/39) and 12% in secondary hospitals (4/33); the difference was statistically significant (*p*<0.001). For hospitals that were not ready for the CR program, the questionnaire addressed the most important obstacles these facilities faced. The most frequently cited problem was the shortage of manpower (32 hospitals), followed by lack of sufficient space (17 hospitals) (Fig 4). Problems with manpower and lack of space accounted for 94% of the total (49 of 52), and there was only one respondent of the opinion that CR was unnecessary. Two hospitals chose the option of “others”; both described non-availability of insurance as a major obstacle. For the 20 hospitals that conducted CR, additional questions about the CR program were surveyed. These hospitals replied that they were conducting programs as follows: educational programs in 17 hospitals; outpatient CR programs in 18; and exercise prescriptions based on cardiopulmonary stress test in 18 (Fig 5). Among the 20 hospitals, 16 hospitals offered all three programs.

## Discussion

This is the first nationwide investigation of CR implementation among hospitals certified for coronary interventions in South Korea. In our study, the overall implementation rate of CR was only 28%. Although the CR implementation rate of tertiary hospitals was higher than that of secondary hospitals, the rate among tertiary hospitals was only 41%, which is still insufficient. Most hospitals without CR programs cited the lack of manpower and space as the biggest obstacles to undertaking CR programs. The Korean Heart Rehabilitation Research Association presented a recommendation for personnel and equipment required for CR in 2010. According to this guideline, medical institutions need a sufficient space of 66 m^2^ or more, including consultation room, examination room, and toilet. It recommended that not only doctors but also nurses, physical therapists, nutritionists, and pharmacists should participate in CR team [12]. That’s why only some large hospitals can satisfy these conditions, and general hospitals will have a burden on high investment costs compared to low reimbursement.

The system of “tertiary medical centers” was implemented in South Korea in 2011 and aims to provide high-quality medical services for severe diseases and establish a healthcare delivery system [13]. The Ministry of Health and Welfare selected excellent medical centers in consideration of their medical performance, educational abilities, manpower, infrastructure, proportion of severe cases, and distribution by region after receiving applications from secondary general hospitals, and a total of 43 hospitals were designated nationwide in 2016. In addition, the Korean Society of Interventional Cardiology evaluated factors such as facilities, manpower, interventional performance, and training abilities. Furthermore, the society certified hospitals above a certain standard as coronary intervention institutes. A total of 93 hospitals were certified as coronary intervention institutes nationwide. We e-mailed every certified coronary intervention institution, including all the tertiary medical centers. Of the hospitals surveyed, 39 hospitals were tertiary centers, accounting for 91% of the total number of tertiary institutions across the country. The hospitals surveyed were distributed across all cities and provinces of the country, with no areas that were not represented in our study (Fig 2). Since our data were obtained from certified coronary intervention hospitals distributed evenly across the country, we believe that our results on the state of CR are generalizable to the whole of South Korea.

The worldwide availability of CR is under 40%, and the rate is much lower in middle- and low-income countries (28% and 8%, respectively) [9]. Even in Japan, which has an advanced medical system, the implementation rate of CR in patients with AMI was reported as only 14–23% in 2007, and the rate of operating outpatient CR programs was critically low at 9% [11]. In a re-investigation five years later, the outpatient CR program operating rate had improved from 9% to 21%, but there is still a shortage compared to the prevalence of AMI [14]. The poor implementation and participation in CR is not an obstacle exclusive to South Korea. Although health policies, economic status, and medical environments vary across different countries, most of the data surveying the status of CR participation have shown disappointing results. In the United States, differences exist from state to state, but the overall CR participation in patients over 65 years of age who received coronary revascularization therapy was about 12.2% by claim-based data of an insurance cohort [15]. In the Netherlands, the CR participation rate was 28% in patients with acute coronary syndrome, stable angina, or chronic heart failure and patients who underwent cardiac surgeries based on a 2007 insurance cohort [16]. In Norway, participation rate was 28% in patients with CHD, based on a 2008–2011 coronary intervention registry [17]. A meta-analysis on the status and clinical effects of CR published in 2016 showed that only 19.4% of the 31 high-income countries had CR participation rates over 50% [18]. The CR participation rate in developing countries is much lower than that in developed countries [19].

CR is underutilized worldwide, but the positive effects of CR in CHD patients have been demonstrated in many previous studies. In CHD patients undergoing revascularization, those who received CR had lower mortality and MI recurrence than those who did not [20]. In addition, CR helps improve mood disorders and exercise capacity and controls risk factors such as weight, blood pressure, and cholesterol [21]. Patients who participated in CR programs also reported higher scores on surveys assessing their quality of life [22]. CR is applicable to patients with reduced functional capacity due to CHD, and CR is also beneficial to all patients who receive revascularization therapy [23]. Although the clinical evidence is not as robust as that available for CHD, CR has been shown to be effective for various heart diseases and issues like valve disease, heart failure, arrhythmia, heart transplantation, and even left ventricular assist devices [24, 25]. Among patients with heart failure with reduced ejection fraction [26], the CR participation group had a lower all-cause mortality and hospital admission rate than controls. Among patients with heart failure with preserved ejection fraction, those who underwent CR had improved exercise tolerance and quality of life [2].

In our study, the lack of manpower and space, the lack of recognition of the necessity of CR, and insurance issues were revealed as obstacles to CR implementation. According to previous studies, barriers that hinder the vigorous implementation of CR can be divided into several categories. The medical staff’s attitude and recognition of the importance of CR are the cornerstone of CR activation [27]. Only one hospital expressed a doubt about the necessity of CR in this survey, and thus the matter of medical staff’s perception is likely not a major cause of CR underutilization in South Korea. Administrative support is another key point and is closely related to the reinforcement of facilities and manpower. Each country has different healthcare policies and insurance-related regulations concerning CR, which have a marked impact on CR implementation. Ireland reported an exceptionally high CR implementation rate (77% of hospitals nationwide in 2003), which is much higher than the rate of only 29% reported in 1998. The poor results of 1998 prompted Ireland to initiate a national cardiovascular health strategy that requires every hospital treating patients with heart disease to provide CR service [28]. The Irish case of rapid growth in the CR implementation rate in a short period proves that administrative support is momentous and effective.

The evidence for mortality and morbidity benefits of CR in patients with CHD is strong. To achieve these potential benefits, however, CR must be implemented widely to ensure adequate reach to everyone who might benefit from it. Our results showed that the implementation of CR programs is low even in certified coronary intervention hospitals. In South Korea, CR was not reimbursed by the NHIS at the time of this study’s enrolment process. Insurance coverage for CR by the NHIS was begun in February 2017. CR implementation was expected to increase significantly, as observed in Ireland, but the results were still disappointing. According to an investigation in 2020, among 164 hospitals performing PCI irrespective of certification by the Korean Society of Interventional Cardiology in South Korea, CR programs were implemented in 47 hospitals (29%) [29]. Compared with the 2016 data, the proportion of CR implementation is still almost the same (28% vs. 29%). Our study targeted only certified hospitals and the later study in 2020 targeted all medical institutions, so direct comparison is inappropriate. However, it was clear that CR implementation rate did not increase than expected after insurance apply. In another investigation, the outpatient CR participation rate of AMI patients from July 2017 to June 2018 was reported as only 1.5% [30]. Even considering that the survey results could be underestimated because of the claim-based data, the CR participation rate of AMI patients is extremely low. There may be various possible reasons why CR utilization has not significantly increased despite insurance coverage being provided by the NHIS. One possibility is that it has only been approximately three years since the insurance coverage began; thus, the effect may not yet be observable. Second, even after starting insurance coverage for CR, the profitability issues remain still because the fee of the CR program determined by NHIS is not large compared to the cost of investment for facilities and manpower. This hurdle could make hospitals reluctant to invest in CR. If CR fees and insurance coverage reach an appropriate level, more hospitals in South Korea will try to implement CR programs. Moreover, insurance coverage for CR is a positive factor in the implementation of CR but it does not mean mandatory. To increase the CR implementation rate at the hospital level, it is reasonable to evaluate the presence of operating CR programs in each hospital and to include them in the hospital quality assessment. This is probably one of the other important ways to enhance CR implementation rate in hospitals.

This study based on a questionnaire survey for each hospital. Therefore, the accuracy of the data is dependent on the respondents. Although questions were asked about whether or not the hospital had a CR program in place, there were no questions about the CR participation rate for AMI patients. Therefore, the actual CR participation rate at the patient level could not obtain in this study. Moreover, in addition to hospitals certified for coronary intervention, at least 30% of other medical institutions treat AMI patients [30]. Most of these institutions without certification for coronary intervention are expected to be smaller and have less staff and equipment for CR than certified centers. Therefore, the real nationwide CR implementation rate at the hospital level in South Korea might be lower than our reported results. However, we originally aimed to investigate CR status in more representative institutions regarding AMI treatment. In addition, this survey was based on hospitals, not individual participants, we did not have information about accessibility to CR facility. However, South Korea is a relatively small country, the hospital accessibility issue is not serious in general.

When the survey was conducted in 2016, there was no provision for NHIS about CR. Health insurance issues may have impacted the activation of CR programs; it is, thus, regrettable that insurance issues were not included in a list of questions pertaining to barriers to CR programs. However, contrary to our expectation, the data from after initiation of insurance coverage still indicate a low rate of CR implementation. Our thought on this finding is the result of a combination of several factors mentioned above. In the Korean medical insurance system, the NHIS and private insurance are used together. If any medical service is covered by the NHIS, the cost of using it is small even if someone does not have private insurance. Therefore, private insurance or patient economic conditions are not obstacles to CR participation in general.

Fortunately, insurance coverage for inpatient and outpatient CR program has been applicable since February 2017. Most of heart disease patients, such as heart failure, ischemic heart disease and cardiac surgery patients are eligible to CR in Korea. It can be guaranteed by NHIS up to twice a day for inpatient and 36 sessions for outpatient. A session is usually 60 minutes long and mainly consists of exercise rehabilitation programs. Generally, the rehabilitation team consists of nurses, physical therapists and nutritionists under the supervision of a doctor. Center based CR is the mainstay in Korea, but community- or clinic-based CR have been rarely introduced. The reason for this is medical institutions should meet the standards for medical staffs, equipment and facilities to receive the CR costs from NHIS, hence it is difficult for small hospitals to initiate CR. Another limitation of this study is that the data is from five years ago. However, our study is still noteworthy because it was conducted only on hospitals that need activation of CR in matter of treating acute myocardial infarction. Also, since insurance coverage for CR began in 2017, this study can present baseline data that can compare before and after insurance coverage.

In conclusion, in contrast to the widespread availability of acute-phase invasive treatment for AMI, the overall implementation of CR is poor in South Korea: the CR utilization rate is better in tertiary than secondary medical centers, but the rates in both facilities need to be improved. Considering the established benefits of CR in AMI patients, urgent efforts (such as sufficient financial support and reflected in hospital quality assessment) should be made to improve this marked underutilization.

## Supporting information

S1 File The full version of questionnaire (English translated version). (DOCX)
