# Supplementary material for: Current status of cardiac rehabilitation among representative hospitals treating acute myocardial infarction in South Korea
Source: PLoS One. 2021 Dec 8;16(12):e0261072. doi: 10.1371/journal.pone.0261072 (PMC8654170; doi:10.1371/journal.pone.0261072)
Supplement: S1 File — (DOCX) [file pone.0261072.s001.docx]

Hospital data

Q 1) Is it a secondary (general) or tertiary hospital? (General/Tertiary)

Q 2) Location of the hospital. (Seoul / Metropolitan cities / Gyeonggi / Gangwon / North Chungcheoung / South Chungcheoung / North Gyeongsang /South Gyeongsang / North Jeolla / South Jeolla / Jeju)

Q 3) Number of hospital beds. (≤299 / 300-499 / 500-999 / ≥1000)

Q 4) Number of cardiologists. (1-2 / 3-5 / ≥6)

Q 5) Presence of an exclusive coronary intensive care unit. (Y/N)

Cardiologic practice data

Q 6) Are AMI patients treated? (Y/N)

Q 7) Is coronary angiography (CAG) performed? (Y/N)

Q 8) Is percutaneous coronary intervention (PCI) performed? (Y/N)

Q 9) Is emergent PCI performed for AMI patients? (Y/N)

Q 10) Is coronary artery bypass graft (CABG) surgery performed? (Y/N)

Implementation of CR

Q 11) Is there a CR program in place? (Y/N)

Q 12) What has been the biggest hurdle in your hospital for starting CR (barriers to CR)? (Understaffed / Lack of space / Not necessary / Others)

If the hospital has been undertaking CR:

Q 13) Is an educational program for AMI available? (Y/N)

Q 14) Is an outpatient CR program available? (Y/N)

Q 15) Is a cardiopulmonary exercise test available? (Y/N)

Q 16) Is a cardiologist in-charge of the CR program? (Y/N)
